# Treatment of canine sarcoptic mange with afoxolaner (NexGard^®^) and afoxolaner plus milbemycin oxime (NexGard Spectra^®^) chewable tablets: efficacy under field conditions in Portugal and Germany

**DOI:** 10.1051/parasite/2018064

**Published:** 2018-12-05

**Authors:** Verena Hampel, Martin Knaus, Jürgen Schäfer, Frederic Beugnet, Steffen Rehbein

**Affiliations:** 1 Conreso GmbH 80331 Munich Germany; 2 Boehringer Ingelheim Vetmedica GmbH, Kathrinenhof Research Center 83101 Rohrdorf Germany; 3 Boehringer Ingelheim Animal Health 69007 Lyon France

**Keywords:** *Sarcoptes scabiei* var. *canis*, afoxolaner, mange, efficacy

## Abstract

The efficacy of NexGard^®^ and NexGard Spectra^®^ against sarcoptic mange in dogs was evaluated in a clinical field study. Skin scrapings from dogs presenting signs suggestive of sarcoptic mange were examined to confirm infestation. A total of 106 dogs were screened at eight sites in Portugal and Germany. In all, 80 dogs that had demonstrated ≥5 live *Sarcoptes* mites in five skin scrapings were enrolled, scored for specific clinical signs (pruritus; papules and crusts; alopecia), and allocated at random to receive either NexGard^®^ or NexGard Spectra^®^ twice, one month apart per label instructions. To determine efficacy, live *Sarcoptes* mites in five skin scrapings per dog were counted, and clinical signs were scored one month and two months after first treatment and compared to pre-treatment (baseline) values. Based on compliance, 65 dogs were determined to be evaluable cases at the end of the study. The efficacy, in terms of reduction of geometric mean live *Sarcoptes* mite counts, was 98.9% and 99.7% for NexGard^®^-treated (*n* = 38) and 99.6% and 100% for NexGard Spectra^®^-treated dogs (*n* = 27) at one month and two months after treatment initiation (*p* < 0.001, both treatments). Both treatments resulted in a significant improvement in pruritus, papules and crusts, and alopecia one month and two months after treatment initiation (*p* = 0.0001, both treatments). In conclusion, this field study confirms that both NexGard^®^ and NexGard Spectra^®^ administered twice one month apart provide an effective and safe treatment against sarcoptic mange in dogs.

## Abstract (fr)

L’efficacité de NexGard^®^ et NexGard Spectra^®^ contre la gale sarcoptique chez le chien a été évaluée dans le cadre d’un essai clinique de terrain. Des raclures cutanées de chiens présentant des signes suspects de gale sarcoptique ont été examinées pour confirmer l’infestation. Au total, 106 chiens ont été dépistés sur huit sites au Portugal et en Allemagne. Quatre-vingt chiens, chez lesquels cinq *Sarcoptes* vivants avaient été détectés dans cinq raclures cutanées, ont été utilisés dans l’étude, examinés pour des signes cliniques spécifiques (prurit, papules et croûtes, et alopécie), et affectés au hasard pour recevoir deux fois NexGard^®^ ou NexGard Spectra^®^, selon les recommandations, à un mois d’intervalle. Pour déterminer l’efficacité, les acariens vivants dans cinq raclures de peau par chien ont été comptés et les signes cliniques ont été notés un mois et deux mois après le premier traitement et comparés aux valeurs avant traitement (valeurs de base). Sur la base de la conformité, il a été déterminé que 65 chiens étaient des cas évaluables à la fin de l’étude. L’efficacité, en termes de réduction des moyennes géométriques d’acariens *Sarcoptes* vivants, était de 98.9 % et 99.7 % pour le traitement par NexGard^®^ (*n* = 38) et de 99.6 % et 100 % pour le traitement par NexGard Spectra^®^ (*n* = 27), à un mois et deux mois après le début du traitement (*p* < 0.001, pour les deux traitements). Les deux traitements ont entraîné une amélioration significative du prurit, des papules et des croûtes, ainsi que de l’alopécie un mois et deux mois après le début du traitement (*p* = 0.0001, pour les deux traitements). En conclusion, cette étude de terrain confirme que NexGard^®^ et NexGard Spectra^®^ administrés deux fois à un mois d’intervalle offrent un traitement efficace et sûr contre la gale sarcoptique chez le chien.

## Introduction

The *Sarcoptes* mites have a permanent association with their hosts and can infest a wide range of mammals, causing scabies in humans and sarcoptic mange in animals. The transmission patterns of *Sarcoptes* mites are not fully understood as this parasite is considered a species comprising several varieties with indistinguishable morphology and different patterns of host specificity [1, 2, 28]. *Sarcoptes* mites recovered from different host species do not usually establish themselves on another. However, *Sarcoptes* mites transferred from animals, including dogs, can infest humans but such infestations (pseudoscabies) are usually mild and self-limiting and disappear without treatment if contact with the source is avoided. The current understanding is that all *Sarcoptes* mites are representatives of one species, *S. scabiei*, which has variants related to host specificity [3, 25].

Canine sarcoptic mange (also referred to as canine scabies or sarcoptic acarosis) is a highly contagious parasitic dermatitis caused by infestation with *S. scabiei* var. *canis* mites. The clinical presentation is characterized by intense pruritus and scratching, alopecia, inflammation, excoriation and hyperkeratosis potentially associated with secondary bacterial infection and pyoderma. Within the range of conditions caused by ectoparasitic mites more commonly seen in dogs (i.e., *Sarcoptes* mange mites, *Otodectes* ear mites, *Demodex* hair follicle mites, *Cheyletiella* fur mites and trombiculid mites), sarcoptic mange and generalized demodicosis are thought to be the most uncomfortable diseases that are able to substantially impair the health and well-being of affected animals. Canine sarcoptic mange is cosmopolitan in occurrence and can affect dogs of all breeds, ages and sexes [18, 20]. In general, it is more frequently seen in not well-nourished dogs or dogs in shelters and kennels but is diagnosed in well-cared-for dogs too, occasionally in association with underlying conditions [12, 13, 18, 20]. In most of Europe, canine sarcoptic mange is considered to be rare but recent data are most often lacking, indicating that the frequency of occurrence is greatly unknown [12]. Recently reported ectoparasite surveys in dogs from south-eastern Europe indicated a prevalence of no more than 5% [24, 32, 36]. However, clinical cases, analyses of dermatological conditions from referral practices or diagnostic laboratories, and parasiticide efficacy studies reported over the past 15 years provide evidence that sarcoptic mange is seen in dogs throughout continental Europe and in the UK [5, 9, 10, 17, 19, 21, 27, 29].

As the health and welfare of affected dogs can be substantially compromised and because of the zoonotic risk for humans in contact with infested dogs, prompt and efficacious treatment is required. Most recently, products containing compounds of the isoxazoline class have been marketed for the treatment and control of flea and tick infestations in dogs. Afoxolaner, an isoxazoline, formulated as chewable tablets either alone (NexGard^®^) or as a fixed combination with the macrocyclic lactone, milbemycin oxime (NexGard Spectra^®^), has been authorized for the treatment and control of infestation in dogs with a range of important ticks and fleas, with monthly dosing [14, 15]. Because of the excellent acaricidal activity with respect to ticks, afoxolaner was evaluated against canine parasitic mite infestations in well-controlled studies and was found to be highly efficacious for the treatment of sarcoptic mange, otoacariosis and demodicosis [6, 8, 11, 22].

The objective of the study reported here was to confirm the efficacy of NexGard^®^ and NexGard Spectra^®^ when administered twice with a monthly interval for the treatment of naturally occurring sarcoptic mange in dogs recruited in veterinary practices.

## Materials and methods

This randomized, multi-center clinical field study was conducted in compliance with VICH GL9, entitled *Good Clinical Practice* and the local animal welfare legislation. The study was performed in a double-blinded manner, i.e., owners as well as all personnel involved in collecting efficacy data and making health observations were masked to the treatment assignment of the animals.

### Study animals

The study animals were recruited by veterinary practices in Portugal and Germany. Client-owned dogs of any breed and sex, with a minimum age of two months and a minimum body weight of 2 kg were eligible for inclusion in the study if they were diagnosed with sarcoptic mange with a minimum of five live *Sarcoptes* mites demonstrated in total five deep skin scrapings collected from lesions suggestive of mange. Dogs intended for breeding or that were pregnant or lactating, dogs demonstrating *Demodex* mites in skin scrapings, dogs receiving immunosuppressive medication including glucocorticoids, or dogs treated with ectoparasiticides (within one month of first intended treatment or during the study) were not eligible for enrolment or were excluded from the study.

In eight veterinary practices, skin scrapings from a total of 106 dogs (Portugal, 103 dogs; Germany, three dogs) presenting pruritus and scratching, alopecia and papulocrustous skin lesions suggestive of sarcoptic mange were examined for causative diagnosis of the condition, and in order to qualify the animals according to the enrolment criteria. Examination of five skin scrapings per dog revealed “no parasitic mites” in the scrapings of seven dogs while *Sarcoptes* and/or *Demodex* mites were demonstrated in the epidermal preparations of 99 dogs. Of these 99 dogs,

- 79 dogs had “≥5 live *Sarcoptes* mites (no concurrent *Demodex* mites)”,
- 14 dogs had “<5 live *Sarcoptes* mites (no concurrent *Demodex* mites)”,
- 4 dogs had “≥5 live *Sarcoptes* mites and concurrent *Demodex* mites”, and
- 2 dogs had “*Demodex* mites (no concurrent *Sarcoptes* mites)”.

Total 80 dogs (Portugal, 78 dogs; Germany, two dogs) were enrolled in the study comprising the 79 dogs which met the inclusion criteria, “≥5 live *Sarcoptes* mites (no concurrent *Demodex* mites)” plus one dog with “≥5 live *Sarcoptes* mites and concurrent *Demodex* mites” which did not meet the study eligibility criteria but was erroneously enrolled. A unique identification number was assigned to each dog, and an Informed Consent and Agreement was obtained from the owners of the dogs or their designees before enrolment. All dogs enrolled in the study underwent a physical examination before first treatment and were considered acceptable for inclusion into the study. Because of non-compliance with study participation criteria or findings during the physical examination before the administration of the second treatment (e.g., no return for second treatment, concurrent use of prohibited medication, pregnancy, lymph node enlargement), 14 dogs were excluded from the study before receiving the second treatment and 66 dogs completed the study. Data for the erroneously enrolled dog, which was kept in the study, were excluded from the data analysis and 65 dogs were considered evaluable cases. These dogs, 36 intact male, one male castrate, 24 intact female and four female spay, ranged in age from approximately two months to 11 years, and weighed 2.0–56.3 kg at enrolment; seven were pedigree dogs (French Bulldog, two; Greyhound, one; Pointer, one; Pug, one; Rottweiler, one; Dachshund, one) and 58 were mixed breed. All dogs remained with their owners, were kept in their usual environments, and received their usual food and water.

### Allocation of dogs to treatment groups and treatment

The study followed a randomized block design, with blocks of two dogs each formed on order of presentation. A unique site-specific randomisation list was provided for each veterinary practice. Within blocks, dogs were allocated to be treated with either afoxolaner chewables (NexGard^®^; Merial, now part of Boehringer Ingelheim) or afoxolaner plus milbemycin oxime chewables (NexGard Spectra^®^; Merial, now part of Boehringer Ingelheim). NexGard^®^ and NexGard Spectra^®^ chewables for oral administration were available in the respective commercial market sizes and were administered to the dogs per the label instructions twice, one month apart (Visit 1, Day 0 and Visit 2, 26–30 days after Visit 2). For dose determination, the dogs were weighed on each day prior to treatment administration.

### Efficacy assessment

To qualify animals for the study based on an etiological diagnosis and for evaluation of efficacy in terms of reduction of live *Sarcoptes* mite counts following treatment, deep skin scrapings (five per dog and per occasion) were collected with a scalpel blade dipped in mineral oil from lesions suggestive of sarcoptic mange, transferred to glass slides which were coverslipped and screened for mites under a compound microscope. Mites were determined based on morphological characteristics [4] and the live (motile) *Sarcoptes* mites were counted. To evaluate efficacy, skin scrapings were obtained and processed as described before prior to first treatment (Visit 1, Day 0), prior to second treatment (Visit 2, 26–30 days after Visit 1), and at study end (Visit 3, 55–61 days after Visit 1).

### Health observations

The dogs received a physical examination by a veterinarian at enrolment (prior to first treatment, Visit 1), prior to the second treatment, and at the end of the study. At each occasion, three clinical signs characteristic for sarcoptic mange were assessed using a three grade score: “Pruritus” (according to the owner’s observation prior to each physical examination) – 0, no (none/virtually no) scratching observed; 1, occasional scratching observed; 2, constant (frequent) scratching observed; “Papules and Crusts” – 0, no papules or crusts; 1, occasional papules or crusts; 2, numerous papules or crusts; “Alopecia” – 0, no alopecia (hair re-growth); 1, areas with alopecia with partial hair (re-)growth; 2, areas with alopecia with no hair re-growth. Dog owners were instructed to report any adverse reaction to the veterinary practice as soon as possible after the reaction was noticed.

At study completion, the veterinarian evaluated the systemic safety based on the occurrence of adverse events throughout the study (Good – no adverse reactions noted; Acceptable – mild and acceptable reactions; Poor – serious and unacceptable reactions).

### Data analysis

Data from all study sites were combined for analysis.

For analysis of the efficacy data, IDV software *Datenanalyse und Versuchsplanung* (Report Version 6.7 and Testimate Version 6.5, validated according to FDA 21 CFR Part 11) was used.

The primary efficacy criterion was the “reduction of live *S. scabiei* var. *canis* mite counts” which was computed comparing each post-treatment count (Visit 2 and Visit 3) with the pre-treatment count (Visit 1, baseline) for each treatment group separately (within group comparison). The percent efficacy of each treatment group was calculated using the formula 100 × [(B − PT)/B], where B is the pre-treatment geometric or arithmetic mean of the treatment group (Visit 1, baseline) and PT is the post-treatment geometric or arithmetic mean of the treatment group at Visit 2 and Visit 3. For the calculation of geometric means, the total number of live mites per animal at each time point was transformed to the natural logarithm of (count +1). To compare the live mite counts of the two time points (Visit 1 vs. Visit 2 and Visit 1 vs. Visit 3) for each treatment group, the Wilcoxon(-Pratt) test was used.

Analysis of the secondary criterion “change of clinical signs”, was computed comparing each post-treatment scoring (Visit 2 and Visit 3) with the pre-treatment scoring (Visit 1, baseline) for each treatment group separately (within group comparison). The Generalized McNemar test for Contingency Tables with Ordered Categories was used to compare the scoring of the two time points (Visit 1 vs. Visit 2 and Visit 1 vs. Visit 3) for each treatment group.

All testing was two-sided at the significance level of *α* = 0.05.

## Results

Sixty-five dogs completed the study per protocol and were evaluable cases, comprising 38 dogs which received NexGard^®^ and 27 dogs which received NexGard Spectra^®^. Prior to treatment, 5–18 live *Sarcoptes* mites were counted in five epidermal preparations obtained from the skin lesions of each of these dogs (Table 1), and all dogs presented pronounced clinical signs characteristic of sarcoptic mange (Table 2).

**Table 1.** *Sarcoptes scabiei* var. *canis* mite counts of naturally infested dogs administered either NexGard^®^ or NexGard Spectra^®^ per label instruction twice, one month apart (Visit 1 and Visit 2) and percentage efficacy of treatments.

		Live *Sarcoptes* mite counts in five skin scrapings		
		Geometric mean^2^ (Range)	Efficacy^3^ (%)	*p*-value^4^
	N+/NG^1^	Arithmetic mean (Standard deviation)		
*NexGard* ^*®*^				
Visit 1, baseline (pre-treatment)	38/38	6.0 (5–18)	–	–
		6.1 (2.67)	–	–
Visit 2, one month after Visit 1	1/38	0.1 (0–10)	98.9	<0.0001
		0.3 (1.62)	95.8	
Visit 3, one month after Visit 2	1/38	<0.1 (0–1)	99.7	<0.0001
		<0.1 (0.16)	99.6	
*NexGard Spectra* ^*®*^				
Visit 1, baseline (pre-treatment)	27/27	6.0 (5–11)	–	–
		6.1 (1.30)	–	–
Visit 2, one month after Visit 1	1/27	<0.1 (0–1)	99.6	<0.0001
		<0.1 (0.19)	99.4	
Visit 3, one month after Visit 2	0/27	0	100	<0.0001
		0	100	

1 N+/NG: Number of dogs tested positive for live *Sarcoptes* mites/Number of dogs examined.

2 Geometric mean, based on transformation to the logarithm of (count + 1).

3 Efficacy (%) = 100 × [(Visit 1 geometric or arithmetic mean mite count − Visit 2 or Visit 3 geometric or arithmetic mean mite count)/Visit 1 geometric or arithmetic mean mite count].

4 Probability from within-group-analysis using Wilcoxon(-Pratt test) at *α* = 0.05.

**Table 2.** Summary and analysis of clinical signs of *Sarcoptes scabiei* var. *canis* naturally infested dogs administered either NexGard^®^ or NexGard Spectra^®^ per label instructions twice, one month apart (Visit 1 and Visit 2).

Clinical sign and score	Number of dogs per score/Number of dogs examined		
	Visit 1, baseline (pre-treatment)	Visit 2, one month after Visit 1	Visit 3, one month after Visit 2
Pruritus			
*NexGard* ^*®*^			
No scratching	0/38	34/38	37/38
Occasional scratching	10/38	4/38	1/38
Constant scratching	28/38	0/38	0/38
*p*-value (Generalized McNemar test)		0.0001	0.0001
*NexGard Spectra* ^*®*^			
No scratching	0/27	24/27	26/27
Occasional scratching	8/27	3/27	1/27
Constant scratching	19/27	0/27	0/27
*p*-value (Generalized McNemar test)		0.0001	0.0001
Papules and crusts			
*NexGard* ^*®*^			
No papules or crusts	0/38	31/38	36/38
Occasional papules or crusts	8/38	6/38	2/38
Numerous papules or crusts	30/38	1/38	0/38
*p*-value (Generalized McNemar test)		0.0001	0.0001
*NexGard Spectra* ^*®*^			
No papules or crusts	0/27	18/27	25/27
Occasional papules or crusts	7/27	9/27	2/27
Numerous papules or crusts	20/27	0/27	0/27
*p*-value (Generalized McNemar test)		0.0001	0.0001
Alopecia			
*NexGard* ^*®*^			
* *No alopecia (hair re-growth)	0/38	21/38	30/38
* *Areas with alopecia with partial hair (re-)growth	6/38	17/38	8/38
* *Areas with alopecia with no hair re-growth	32/38	0/38	0/38
* p*-value (Generalized McNemar test)		0.0001	0.0001
*NexGard Spectra* ^*®*^			
* *No alopecia (hair re-growth)	0/27	9/27	20/27
* *Areas with alopecia with partial hair (re-)growth	3/27	17/27	7/27
* *Areas with alopecia with no hair re-growth	24/27	1/27	0/27
* p*-value (Generalized McNemar test)		0.0001	0.0001

Compared to baseline, live *Sarcoptes* mite counts, based on geometric means, were significantly reduced by >98% in both treatment groups following the first treatment (Visit 2 examination). Following administration of two treatments, one month apart, 37 of the 38 dogs treated with NexGard^®^ and all of the 27 dogs that received NexGard Spectra^®^ had no live *Sarcoptes* mites in the skin scrapings obtained at the study end (Visit 3), which translates into >99% and 100% reduction of live *Sarcoptes* mite counts relative to pre-treatment, respectively (Table 1).

**Figure 1.**
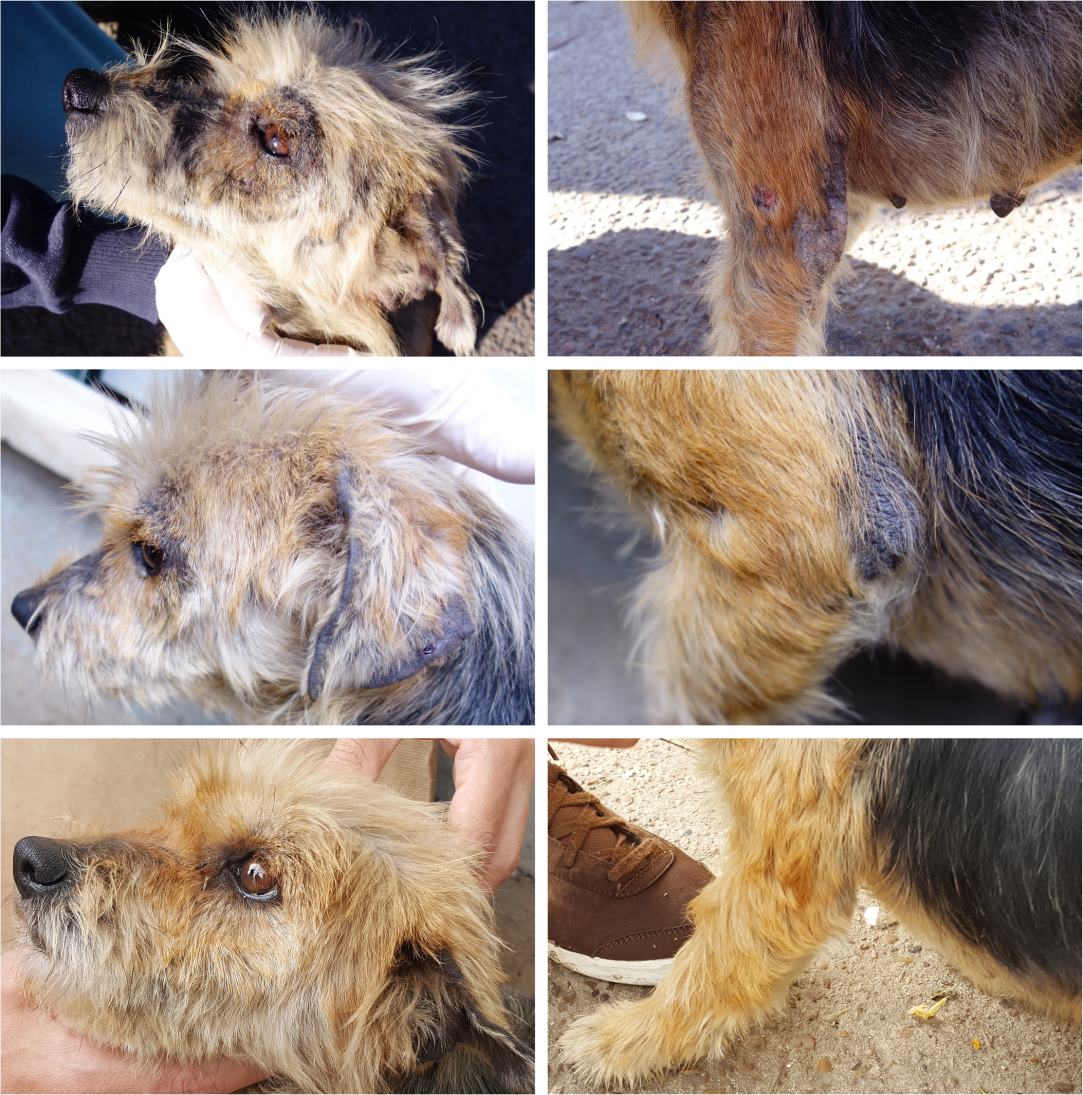
Dog with sarcoptic mange pre-treatment (first row pictures), and one month (second row pictures) and two months (third row pictures) after initiation of treatment with NexGard^®^.

**Figure 2.**
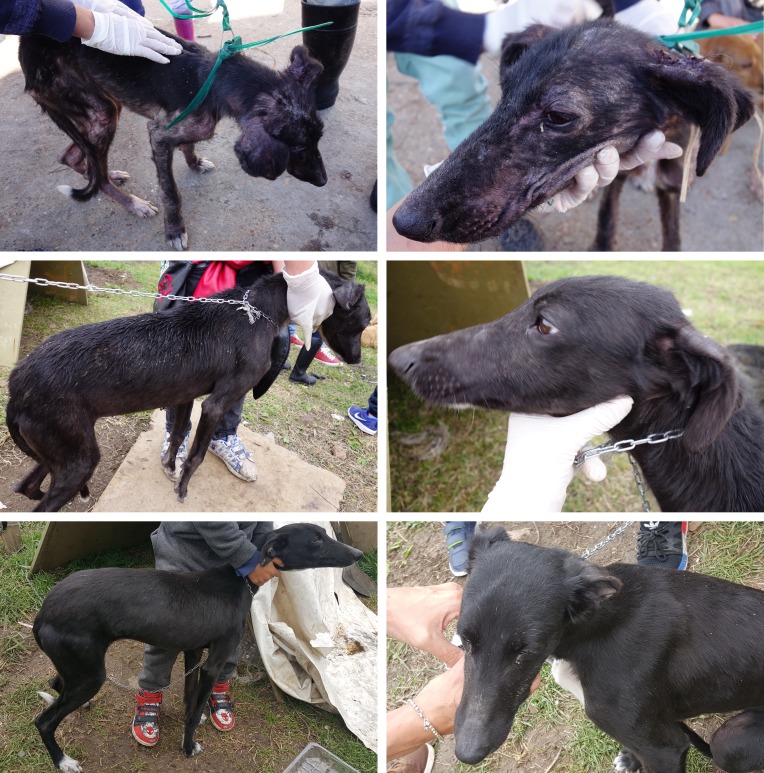
Dog with sarcoptic mange pre-treatment (first row pictures), and one month (second row pictures) and two months (third row pictures) after initiation of treatment with NexGard Spectra^®^.

Compared to baseline, all clinical signs improved significantly in response to the treatment with both NexGard^®^ and NexGard Spectra^®^ over the two month observation period. The clinical signs had substantially improved one month after the first treatment already, and further improvement was recorded one month after the second treatment. After two monthly treatments with either NexGard^®^ or NexGard Spectra^®^, “Pruritus” was completely resolved in all dogs except one in each group which was reported occasionally scratching, and “Papules and Crusts” were completely resolved in all dogs except two in each group which presented occasional papules or crusts. While all dogs had “Alopecia” pre-treatment, all dogs treated with NexGard^®^ and NexGard Spectra^®^ had hair re-growth at study completion, with 30 and 20 dogs presenting no remaining alopecia, and eight and seven presenting areas with some alopecia and hair re-growth, respectively (Table 2). Colored pictures taken at each examination of two dogs treated with NexGard^®^ or NexGard Spectra^®^ are shown in Figures 1 and 2 to illustrate the clinical recovery.

No treatment-related adverse experiences were observed in any dogs that completed the study.

## Discussion

Parasitic infestation-related conditions, bacterial infections and allergic skin diseases constitute the most common dermatological diagnoses reported in dogs. Among dermatological cases collected from the records of 20 general veterinary practices in the UK, flea infestation/allergy, mite infestation (trombiculosis, scabies, demodicosis, otoacariosis) and ticks were diagnosed in decreasing order of frequency [19].

The examination of deep skin scrapings from the 106 dogs that presented with clinical signs typical of sarcoptic mange, and that were screened for enrolment into the study, demonstrated *Sarcoptes* mites as the etiologic agent in more than 90% of the dogs. This indicates that the concurrent presentation in dogs of pruritus, crusted papule skin lesions and alopecia are strongly suggestive of sarcoptic mange and allow for fairly reliable clinical diagnosis [17]. However, the suspected clinical diagnosis must be confirmed in order to initiate an adequate treatment procedure. The definitive diagnosis of sarcoptic mange relies on the demonstration of *Sarcoptes* mites in skin scrapings [13].

Several acaricidal products have been licensed for the treatment of *S. scabiei* infestation in dogs, with topically administered locally acting products representing the more traditionally used treatments, while topical products providing systemic acaricidal activity or a combination of local and systemic activity have been authorized within the last decade. The most recently commercialized ectoparasiticides of the isoxazoline class of compounds provide highly efficacious and very convenient treatments of ectoparasite infestations through systemic exposure of the parasites following administration of the active substances in chewable tablet and/or topical formulations.

The results of the study reported here demonstrated the high efficacy of afoxolaner and afoxolaner plus milbemycin oxime oral chewable tablets (NexGard^®^ and NexGard Spectra^®^, respectively) against sarcoptic mange under practical use conditions, as previously reported in well-controlled studies for three isoxazoline-based products including afoxolaner [5, 6, 33]. The rapid scabicidal effect of the treatment with NexGard^®^ and NexGard Spectra^®^ was associated with gradual improvement of the clinical signs in all dogs. Treatment of the dogs resulted in substantial improvement of all clinical signs of sarcoptic mange and was statistically significant within one month subsequent to the initial treatment. Consistent with findings reported in other studies which evaluated the efficacy of oral isoxazoline products against canine sarcoptic mange, pruritus has been the clinical sign that completely resolved in nearly all treated dogs, while mild papulocrustous skin lesions and alopecia may be observed in some animals for a longer period, potentially related to prolonged contact with mite-derived material in or on the skin [5, 6, 33]. The adjunct use of non-medicated bathing or shampooing may be of benefit for the clinical management of the skin condition and may accelerate the complete resolution of the skin conditions [7].

In the present field efficacy study, afoxolaner (NexGard^®^) and afoxolaner plus milbemycin oxime (NexGard Spectra^®^) oral chewable tablets demonstrated high efficacy against canine sarcoptic mange, as was previously shown in the evaluation of the two products against infestation with *Dermacentor reticulatus* ticks [31]. With milbemycin oxime added to afoxolaner in order to extend the ectoparasiticidal spectrum of NexGard^®^ to the treatment of intestinal nematode infections [16, 30, 34], and to the prevention of heartworm disease and *Angiostrongylus vasorum* lungworm infection [23, 35], NexGard Spectra^®^ chewable tablets contain two compounds with miticidal activity. However, in order to achieve therapeutic efficacy against sarcoptic mange, milbemycin oxime requires frequent repeated oral administration at elevated dosages [26]. In the fixed combination with afoxolaner in NexGard Spectra^®^, which was demonstrated to be highly efficacious against sarcoptic mange when administered per the label instructions one month apart, the milbemycin oxime dose is too low to account for meaningful scabicidal activity.

Authorized products that have been proven to be safe and that are both convenient to administer and efficacious are preferred by practitioners and dog owners. Whichever therapeutic regimen is used, it is crucial to treat all dogs known to have been in contact with an affected animal to reduce the risk of potential re-infestation from these sources [13].

The results of the present study under practical use conditions demonstrated that both afoxolaner and afoxolaner plus milbemycin oxime chewable tablets (NexGard^®^ and NexGard Spectra^®^, respectively) are highly effective and safe treatments of canine sarcoptic mange when administered twice, one month apart, and that both treatments are associated with rapid improvement in the clinical signs of this important parasitic skin disease. The regular use of isoxazoline products for the control of flea and tick infestations, or isoxazoline/anthelmintic combination products for the control of flea and tick infestations plus treatment of gastrointestinal nematode infections and prevention of heartworm disease and lungworm infection, provides effective treatment of canine sarcoptic mange and can be expected to prevent *Sarcoptes* mite infestation.
